# Generation and characterization of ABT-981, a dual variable domain immunoglobulin (DVD-Ig^TM^) molecule that specifically and potently neutralizes both IL-1α and IL-1β 

**DOI:** 10.1080/19420862.2015.1026501

**Published:** 2015-03-12

**Authors:** Susan E Lacy, Chengbin Wu, Dominic J Ambrosi, Chung-Ming Hsieh, Sahana Bose, Renee Miller, Donna M Conlon, Edit Tarcsa, Ravi Chari, Tariq Ghayur, Rajesh V Kamath

**Affiliations:** 1 AbbVie Bioresearch Center; Global Biologics; Worcester, MA USA; 2 Shanghai CP Guojian Pharmaceutical Co., Ltd.; Shanghai, China; 3 AbbVie Bioresearch Center; Global Biologics; Worcester, MA USA; 4 AbbVie Bioresearch Center; Immunology Pharmacology; Worcester, MA USA; 5 AbbVie Bioresearch Center; DMPK-BA; Worcester, MA USA; 6 AbbVie Bioresearch Center; Drug Product Development; Worcester, MA USA; 7 AbbVie Bioresearch Center; Foundational Immunology; Worcester, MA USA

**Keywords:** IL-1α, IL-1β, osteoarthritis, DVD-Ig, ABT-981, IL-1 antagonist

## Abstract

Interleukin-1 (IL-1) cytokines such as IL-1α, IL-1β, and IL-1Ra contribute to immune regulation and inflammatory processes by exerting a wide range of cellular responses, including expression of cytokines and chemokines, matrix metalloproteinases, and nitric oxide synthetase. IL-1α and IL-1β bind to IL-1R1 complexed to the IL-1 receptor accessory protein and induce similar physiological effects. Preclinical and clinical studies provide significant evidence for the role of IL-1 in the pathogenesis of osteoarthritis (OA), including cartilage degradation, bone sclerosis, and synovial proliferation. Here, we describe the generation and characterization of ABT-981, a dual variable domain immunoglobulin (DVD-Ig) of the IgG1/k subtype that specifically and potently neutralizes IL-1α and IL-1β. In ABT-981, the IL-1β variable domain resides in the outer domain of the DVD-Ig, whereas the IL-1α variable domain is located in the inner position. ABT-981 specifically binds to IL-1α and IL-1β, and is physically capable of binding 2 human IL-1α and 2 human IL-1β molecules simultaneously. Single-dose intravenous and subcutaneous pharmacokinetics studies indicate that ABT-981 has a half-life of 8.0 to 10.4 d in cynomolgus monkey and 10.0 to 20.3 d in rodents. ABT-981 exhibits suitable drug-like-properties including affinity, potency, specificity, half-life, and stability for evaluation in human clinical trials. ABT-981 offers an exciting new approach for the treatment of OA, potentially addressing both disease modification and symptom relief as a disease-modifying OA drug.

## Abbreviations

ADA: Anti-drug antibodies
CDR: complementarity-determining region
CEC: cation exchange chromatography
Cyno: cynomolgus monkey
DVD-Ig: dual variable domain immunoglobulin
ED_50_: efficacious dose at 50% response
ED_80_: efficacious dose at 80% response
ELISA: enzyme-linked immunosorbent assay
IL-1: interleukin-1
IgG1: immunoglobulin G1
IV: intravenous
OA: osteoarthritis
PAS: protein A sepharose
SC: subcutaneous
SEC: size exclusion chromatography
SPR: surface plasmon resonance
V_H_: antibody heavy chain variable domain
V_L_: antibody light chain variable domain

## Introduction

The interleukin (IL)-1 superfamily includes 7 agonist proteins (including IL-1α and IL-1β), 3 receptor antagonists (including IL-1Ra) and the anti-inflammatory cytokine IL-37.^1^ IL-1α and IL-1β are distinct pro-inflammatory cytokines produced by multiple cell types, e.g., monocytes, macrophages, and neutrophils, as well as by cells in joint tissues e.g., synovial fibroblasts, chondrocytes and osteoclasts.^2-4^ They exert a wide range of biological and physiological effects throughout the body, including fever following stimulation in the central nervous system, innate resistance to infection,^5^ prostaglandin synthesis,^6^ T- and B-lymphocyte activation, IL-2 production, expression of cytokines, chemokines, nitric oxide synthetase, and matrix metalloproteinases (MMPs).^7^ IL-1α and IL-1β bind to the same receptor, namely the IL-1 receptor type I (IL-1R1) complexed to the IL-1 receptor accessory protein (IL-1RAcP) and induce similar physiological effects, including neutrophilia, fibroblast proliferation, cytotoxicity for certain cells, induction of collagenases, and increased production of colony stimulating factors and collagen.^5,8^ The effects of IL-1 are negatively regulated by 2 endogenous inhibitors, namely IL-1 receptor 2 (IL-1R2), which is related to the IL-1R1 and lacks a cytoplasmic domain, and IL-1 receptor antagonist IL-1Ra, which mimics the binding of IL-1β to IL-1R1 but fails to recruit the IL-1RAcP, preventing downstream signaling.^5^

Both IL-1α and IL-1β are produced as precursor proteins that are proteolytically processed to mature forms. The IL-1α precursor is active and constitutively expressed in the epithelium of many organs and tissues, and is released following necrotic cell death.^9,10^ Mature IL-1α is expressed both as a soluble and membrane form, and it is thought to act locally.^11-14^ The IL-1β precursor, which is synthesized in hematopoetic cells, is inactive until it is cleaved by caspase-1, releasing the mature IL-1β into circulation.^13^ Overexpression of human IL-1α in mice results in chronic inflammatory arthritis that is macrophage and neutrophil-dominant,^15^ and mice that selectively overexpress the mature membrane-associated human IL-1α display a more severe arthritis characterized by autonomous synovial proliferation with subsequent cartilage destruction.^16^ Intra-articular injection of IL-1β results in a reproducible acute model of joint pathology, including swelling and hyperalgesia.^17^ In rats, injection of recombinant IL-1β results in persistent pain ^18^ and increased sensitivity to pain.^19^

Osteoarthritis (OA) is a disease of the whole joint with the signature pathologic feature of articular cartilage loss leading to joint destruction. The clinical symptoms of OA are pain and functional impairment that includes joint stiffness and dysfunction. In recent years, it has become increasingly apparent that a complex cascade of mechanical and biochemical pathways contributes to the initiation, maintenance and progression of the disease.^2^ Preclinical and clinical studies provide significant evidence for the role of IL-1 in the pathogenesis of OA, including cartilage degradation, bone sclerosis, and synovial proliferation.^20^ IL-1α and IL-1β are expressed in the cartilage and synovial membrane and fluid of patients with OA.^7,21,^^22^ Members of the IL-1 family can stimulate migration and activation of neutrophils,^7^ which are the most abundant cell infiltrate when acute synovitis occurs in patients with OA.^23^ IL-1 can stimulate chondrocytes and synoviocytes to produce proteinases involved in cartilage destruction leading to OA ^24-26^ as well as inhibit synthesis of proteoglycan and collagen type II, the main components of the extracellular matrix (ECM) in articular cartilage.^27,28^ Therefore, IL-1 not only causes degradation of cartilage, but also suppresses reparative processes. Intra-articular injection of IL-1 into animal knees results in leukocyte infiltration and cartilage loss,^29^ and intra-articular injection of IL-1 antagonist results in significant reduction in the progression of experimental OA.^30-33^ Gene expression studies using peripheral blood leukocytes (PBLs) from OA patients showed patients with overexpression of IL-1β had higher pain scores, decreased function, and were at a higher risk of radiographic progression of OA.^34^ Finally, IL-1 knockout (KO) mice are resistant to surgically induced cartilage damage compared to their wild‑type counterparts, as well as development of allodynia in inflammatory and neuropathic models of pain.^35,36^

Therapeutic agents that interfere with the IL-1 pathway have been tested clinically. AMG 108, a IL-1R1-targeted human IgG2 monoclonal antibody (mAb), was evaluated intravenously (IV) and subcutaneously (SC) in patients with knee OA.^37^ AMG-108-dosed patients showed statistically insignificant improvements in pain, although numerically greater improvements were observed. Anakinra (a recombinant form of native IL-1Ra) was evaluated in an intra-articular study in patients with painful knee OA ^38^ and with symptomatic OA of the knee.^39^ Although anakinra was well-tolerated in patients from this study, anakinra treatment did not result in improvements in OA symptoms compared to placebo. Given the results from these clinical studies, clinicians have suggested future IL-1 pathway inhibitors be designed to allow evaluation of a more potent IL-1 inhibitor with a longer duration.^39^ A more potent inhibitor of the IL-1 pathway would neutralize the activity of both IL-1α and IL-1β, but would not interfere with the natural antagonist, IL-1Ra. In addition, a more potent inhibitor would achieve a longer duration if it was a molecule more comparable to the structure and molecular mass of an immunoglobulin. The generation of such molecules for preclinical proof-of-concept studies, namely chimeric anti-IL-1α/β dual variable domain immunoglobulins (DVD-Ig) composed of mouse mAb variable domains with human constant regions, has been described.^40^ In addition, murine DVD-Igs have been generated and evaluated in a mouse model of OA, wherein simultaneous inhibition of mouse IL-1α and IL-1β reduced OA progression^41^ and pain^42^ to a greater extent than inhibition of either IL-1 isoform alone. Despite the successful generation of chimeric and murine DVD-Igs, the murine variable domains used to construct these molecules were not adequate to generate a human DVD-Ig that demonstrates the rigorous drug-like properties required of a clinical-grade therapeutic protein. Here, we describe the generation and characterization of ABT-981, a human anti-IL-1α/β therapeutic DVD-Ig now in clinical trials that offers an exciting new approach for the treatment of OA, potentially addressing both disease modification and symptom relief as a disease-modifying OA drug (DMOAD).

## Results

### Identification of anti-human IL-1α and IL-1β antibodies

To engineer a therapeutic human DVD-Ig capable of potently neutralizing both human IL-1α and IL-1β, several antibody variable domain sequences were considered. In addition to neutralization of human IL-1α and IL-1β, neutralization of cynomolgus IL-1α and IL-1β was also required to allow the toxicological evaluation of the resulting human DVD-Ig molecules in cynomolgus monkeys. Heavy and light chain antibody variable domain sequences specific for human IL‑1α were derived from the human auto-antibody X3, originally isolated from a human B cell library.^43^ Heavy and light chain antibody variable domain sequences specific for human IL‑1β were derived from the humanized SK48-E26 antibody.^44^ cDNAs corresponding to the published antibody sequences of X3 and SK48-E26 were synthesized (Blue Heron, Bothell, WA) and expressed as human IgG1/k mAbs for characterization studies.

### Characterization of mAb X3

**Figure 1.**
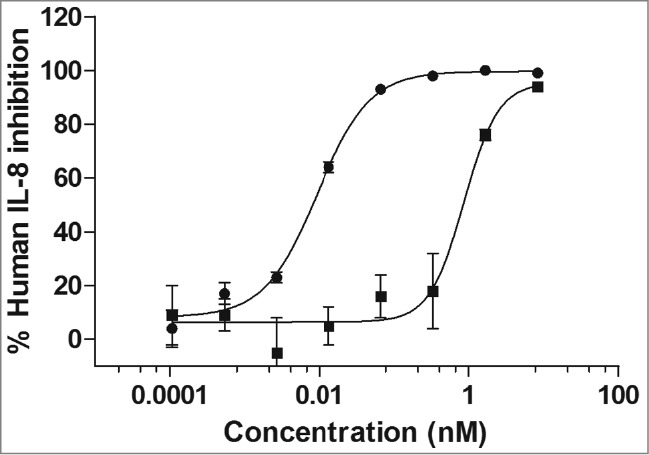
Potency determination of mAb X3 on human and cynomolgus IL-1α. The MRC-5 cell line produces IL-8 in response to stimulation with both human and cynomolgus IL-1α and IL-1β. In this potency assay, MRC-5 cells were stimulated with 3 pM of either human (•) or cynomolgus (▪) IL-1α in the presence of increasing concentrations of the IL-1α-specific mAb X3.

Following the synthesis and expression of the X3 mAb cDNA, binding and potency characterizations were carried out. The binding affinity data generated by surface plasmon resonance (SPR) demonstrated the affinity of the X3 mAb was ∼20-fold less to cynomolgus IL-1α compared to human IL-1α (**Table 1**). To understand if this affinity difference had an effect on the neutralization potency of the X3 mAb, a potency assay using MRC-5 cells was performed. The MRC-5 cell line (human lung fibroblast) produces IL-8 in response to stimulation with both human and cynomolgus IL-1α and IL-1β, and this response is inhibited in the presence of a neutralizing antibody. The MRC-5 bioassay results showed that the neutralization potency of mAb X3 to human versus cynomolgus monkey IL-1α differed by ∼100-fold, indicating that the lower affinity of the mAb to cynomolgus IL-1α resulted in less neutralization potency (**Fig. 1** and **Table 1**). Of note, additional characterization studies determined that the X3 mAb did not neutralize murine, rat, canine or rabbit IL-1α (data not shown).

**Table 1.** Potency and affinity characterization of mAb X3

Binding affinity K_D_ (pM)		Neutralization potency IC_50_ (pM)	
Human IL-1α	Cynomolgus IL-1α	Human IL-1α	Cynomolgus IL-1α
20	417	9	862

### Generation and characterization of affinity-matured humanized IL-1β antibodies

The humanized SK48-E26 mAb (herein referred to as SK48-E26) was expressed, characterized in the MRC-5 potency assay, and affinity matured using yeast display technology. Five scFv yeast display libraries containing mutations in the complementarity-determining region (CDR) regions of the SK48-E26 heavy and light chain variable domains were generated to allow isolation of clones that bound with higher affinity to both biotinylated human and cynomolgus IL-1β. Following isolation and sequence analysis of scFv yeast display clones, 6 affinity-matured heavy chain and 2 affinity-matured light chain variable domains were selected based on sequence changes and prevalence in the outputs. Several affinity-matured SK48-E26 variant human IgG1/k antibodies (E26.1, E26.2, E26.12, E26.13, E26.35 and E26.37) were generated by expression of a specific affinity-matured variable heavy chain (V_H_) sequence (E26.1 V_H_, E26.2 V_H_, E26.12 V_H_, E26.13, V_H_ E26.35 V_H_ and E26.37 V_H_) paired with the SK48-E26 variable light chain (V_L_) sequence (SK48-E26 V_L_) or the corresponding affinity-matured variable light chain sequence (E26.1 V_L_ and E26.37 V_L_). The mutations present in the variable domain of each of these variants compared to the humanized SK48-E26 sequence are highlighted in **Figure S1**. Following purification, each affinity-matured variant was evaluated and characterized in assays, including binding to human IL-1β by ELISA and SPR, and neutralization potency to human IL-1β by MRC-5 cell bioassay (**Table 2**). Although all mAbs were analyzed for binding to human IL-1β by ELISA, only a subset were subjected to kinetic and bioactivity analysis. Overall, mAbs E26.13 and E26.35 were fully characterized across all parameters. Of note, additional characterization studies determined that the E26.13 mAb did not neutralize murine, rat or canine IL-1β (data not shown).

**Table 2.** Characterization of affinity-matured versions of SK48-E26

	Binding affinity EC_50_	Binding affinity K_D_		Neutralization potency IC_50_	
Antibody name	Human IL-1β (pM)	Human IL-1β (pM)	Cyno IL-1β (pM)	Human IL-1β (pM)	Cyno IL-1β (pM)
SK48-E26	ND	242	ND	400	185
E26.1	12.9	ND	ND	9.7	ND
E26.2	567	52.8	ND	ND	ND
E26.12	306	78.6	ND	ND	ND
E26.13	7.2	44.5	14.6	15.7	8.4
E26.35	10.4	23.9	14	7.2	3
E26.37	17.7	ND	ND	2.2	ND

**Legend:** Binding affinity EC_50_ data were generated using an ELISA based on the detection of biotinylated IL-1β binding captured mAb (via an anti-human IgG Fc antibody coated on the ELISA plate). Binding affinity (K_D_) data were generated by surface plasmon resonance using a Biacore 3000 instrument based on the detection of human or cynomolgus IL-1β binding to captured mAb (via a goat anti-human IgG Fc coated on the CM5 biosensor chip). Neutralization potency IC_50_ was generated using an MRC-5 cell bioassay in which 3 pM of human or cynomolgus IL-1β stimulation resulted in the release of human IL-8. Human IL-8 in cell supernatants was quantified using a human IL-8 ELISA. ND: Not determined.

### Generation of human IL-1α/β DVD-Ig™ molecules

**Figure 2.**
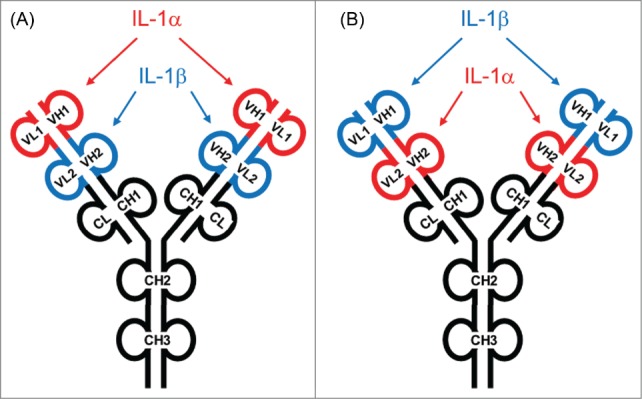
Design of human IL-1α/β dual variable domain immunoglobulins (DVD-Igs). (**A**) Representation of a DVD-Ig with an anti-IL-1α variable domain on the “outer” position linked to an anti-IL-1β variable domain in the “inner” position. (**B**) Representation of a DVD-Ig with an anti-IL-1β variable domain in the “outside” position linked to an anti-IL-1α variable domain in the “inner” position. Several DVD-Igs were constructed, expressed in HEK 293 cells, and purified from cell supernatants using Protein A affinity chromatography. The architecture of ABT-981 is based on design **B**.

Several human DVD-Igs were generated and characterized using V_H_ and V_L_ amino acid sequences from the human IL-1α-specific autoantibody X3 and affinity-matured V_H_ and V_L_ amino acid sequences from affinity-matured IL‑1β-specific mAbs E26.13 and E26.35. In terms of domain orientation, some cDNAs encoding DVD-Ig heavy and light chains were assembled with the IL‑1β binding domains in the “outer” position and the IL‑1α binding domains in the “inner” position (**Fig. 2**). Other DVD-Igs were constructed with cDNAs encoding heavy and light chains assembled with the IL‑1α binding domains in the “outer” position and the IL‑1β binding domains in the “inner” position. The position of each domain is indicated in the associated DVD-Ig nomenclature, namely, outer variable domain – linker – inner variable domain (for example, the E26.13-LL-X3 DVD-Ig is comprised of the IL-1β mAb E26.13 in the outer domain, an L linker in the heavy chain, an L linker in the light chain, and the IL-1α mAb X3 in the inner domain). In the case of linker designation “LL," the outer and inner heavy chain antibody variable domain sequences were fused in-frame via nucleotides encoding the human linker amino acid sequences ASTKGPSVFPLAP and the light chain antibody variable sequences were fused in-frame via nucleotides encoding the human linker amino acid sequence TVAAPSVFIFPP.^45^ In the case of the linker designation “SS," the outer and inner heavy chain antibody variable domain sequences were fused in-frame via nucleotides encoding the human linker amino acid sequences ASTKGP and the light chain antibody variable sequences were fused in-frame via nucleotides encoding the human linker amino acid sequence TVAAP.^45^

Each DVD-Ig heavy chain was expressed with a human IgG1 constant domain containing leucine-to-alanine amino acid substitutions in the hinge region at Kabat numbering positions 364 and 365 (in an IgG1 molecule these positions are 234 and 235). Variable domain cDNA sequences in the DVD-Ig light chains were expressed with a human kappa constant domain. The IgG1 constant region amino acid hinge region mutations were incorporated to eliminate the effector function of the IgG1 by altering its interactions with Fcγ receptors. Compared to wild-type IgG1/k mAbs, the IgG1 mAbs containing the L234A, L235A hinge-region mutation demonstrated several features when studied in vitro, namely, retained binding to FcR_N_ in a concentration- and pH-dependent manner and undetectable binding to FcγRI, FcγRIIb, FcγRIIIa, and complement C1q (data not shown). Combinations of human heavy chain- and light chain-expressing plasmids were expressed in HEK293 cells and DVD-Igs were purified from cell supernatants by protein A affinity chromatography.

### Characterization of IL-1α/β DVD-Igs

To understand the consequences of variable domain sequence and orientation within the DVD-Ig structure, the IL-1α/β DVD-Igs were characterized for affinity to IL-1α and IL-1β by ELISA and SPR (Biacore) (**Table 3**), and neutralization potency using an MRC-5 cell bioassay (**Table 3**). In terms of expression levels, the DVD-Igs having the IL-1β variable domain in the outer position (E26.13-LL-X3, E26.13-SS-X3, E26.35-SS-X3) expressed more protein than the DVD-Igs with the IL-1β variable domain in the inner position (X3-LL-E26.13 and X3-SS-E26.13) (data not shown). As a result, not all molecules were thoroughly characterized in all assays. In terms of observed trends in affinity, the IL-1β mAb E26.13 had an EC_50_ of 7.2 pM (**Table 2**) and the same variable domain expressed in the outer domain of 2 DVD-Igs (E26.13-LL-X3, E26.13-SS-X3) demonstrated a comparable EC_50_ range of 7.5–8.1 pM (**Table 3**). These data suggest that neither the outer domain position nor the LL vs. SS linker had an effect on the 3-dimensional folding of the E26.13 variable domain as it was fused to the IL-1α variable domain X3 in the inner position. However, the EC_50_ shifted to 70 pM when this same variable domain was expressed on the inner domain of 2 DVD-Igs in which the IL-1α mAb X3 was positioned in the outer domain (X3-LL-E26.13 and X3-SS-E26.13). By SPR, the overall affinity (K_D_) of the E26.13 mAb to IL-1β was ∼45 pM (**Table 2**), and when expressed in the outer position in 3 DVD-Igs, the overall affinity (K_D_) of the E26.13 variable domain ranged from 7.8–21 pM (**Table 3**). These binding data suggest the 3-dimensional structure of the E26.13 variable domain was not optimally preserved in the inner position of the DVD-Ig format compared to the outer position or as a mAb. In terms of potency in the MRC-5 bioassay, the E26.13 mAb ranged from 8.4 to 15.7 pM to both human and cynomolgus monkey (**Table 2**), and this range was preserved when the variable domain was expressed in the outer domain of the DVD-Ig (8.4 to 18.3 pM,**Table 3**). When expressed in the inner domain in the DVD-Ig, E26.13 showed a potency range of 1470 to 2676 to human IL-1β, suggesting that, in conjunction with these binding data, the architecture of the E26.13 variable domain was not optimally preserved in the inner domain of the DVD-Ig format (**Table 3**).

**Table 3.** Binding affinity and neutralization potency of IL-1α/β DVD-Igs to human and cynomolgus IL-1α and IL-1β

	Binding affinity EC_50_ (pM)		Binding affinity K_D_ (pM)				Neutralization potency IC_50_ (pM)			
	Human		Human		Cyno		Human		Cyno	
DVD-Ig	IL-1β	IL-1α	IL-1β	IL-1α	IL-1β	IL-1α	IL-1β	IL-1α	IL-1β	IL-1α
E26.13-LL-X3	8.1	5.8	7.8	6.2	12.4	324	18.3	10.2	16.7	1053
E26.13-SS-X3	7.5	6.4	21	7.6	15.3	411	16.0	16.2	8.4	955
E26.35-SS-X3	8	6.2	5	13	10.6	327	1.8	25.8	0.6	474
X3-LL-E26.13	ND	ND	ND	ND	ND	ND	1470	8.9	ND	ND
X3-SS-E26.13	70	4.5	ND	ND	ND	ND	2676	7.6	ND	ND

**Legend:** Binding affinity EC_50_ data was generated using an ELISA based on the detection of biotinylated IL-1α or IL-1β binding to captured DVD-Igs (captured via an anti-human IgG Fc antibody coated on the ELISA plate). Binding affinity (K_D_) data were generated by surface plasmon resonance using a Biacore 3000 instrument based on the detection of human or cynomolgus IL-1β or human or cynomolgus IL-1α binding to captured mAb (via a goat anti-human IgG Fc coated on the CM5 biosensor chip). The DVD-Ig nomenclature is outer domain – linker – inner domain. Neutralization potency IC_50_ was generated using an MRC-5 cell bioassay in which 3 pM of human or cynomolgus IL-1α or IL-1β stimulation resulted in the release of human IL-8. Human IL-8 in cell supernatants was quantified using a human IL-8 ELISA. ND: Not determined.

The IL-1β antibody E26.35 maintained affinity in the outer position of DVD-Ig E26.35-SS-X3 (10.4 pM in the monoclonal version compared to 8 pM in the DVD-Ig by ELISA; **Table 2** and **Table 3**). Compared to the monoclonal version, the overall affinity of the E26.35 domain to both human and cynomolgus monkey IL-1β in the outer position of the DVD-Ig increased from 23.9 pM to 13 pM on human IL-1β and from 14 pM to 10.6 pM on cynomolgus monkey IL-1β (**Table 2**). In terms of potency in the MRC-5 bioassay, the E26.35 mAb demonstrated a potency range of 3 to 7.2 pM on human and cynomolgus IL-1β. Once in the outer position of the DVD-Ig format, this variable domain showed an increase in potency to both human and cynomolgus IL-1β, with a potency range of 0.6 to 1.8 pM (**Table 3**).

The overall affinity (K_D_) of the X3 variable domain to human IL-1α when positioned in the inner domain of 3 DVD-Igs was 6.2 to 13 pM (**Table 3**), comparable to the 20 pM K_D_ affinity determined with the X3 as a mAb (**Table 1**). The K_D_ of the X3 variable domain to cynomolgus IL-1α when positioned in the inner domain ranged from 324 to 411 pM (**Table 3**), a value comparable to the monoclonal version of X3, suggesting no negative impact of DVD-Ig domain position on the neutralization potency. When in the outer domain position of 2 DVD-Igs, the X3 variable domain IC_50_ was 7.6 pM, compared to 9 pM when expressed as a mAb. Overall, data from the characterization studies indicates the IL-1β variable domains E26.13 and E26.35 retain their respective affinities and potencies when positioned in the outer domain of the DVD-Ig, whereas the IL-1α variable domain X3 is more tolerant to being in either the inner or outer position. Based on affinity and potency data, the 197 kDa DVD-Ig referred to as E26.13-SS-X3 was selected for further characterization studies, and it was re-named ABT-981.

### Consecutive binding of IL-1α/β to ABT-981

**Figure 3.**
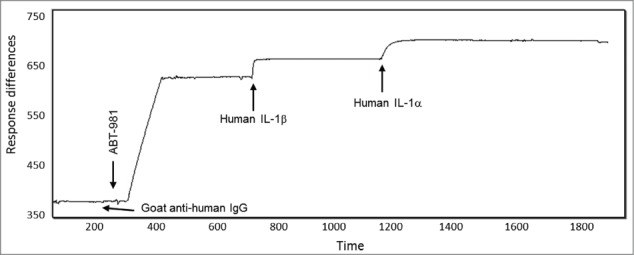
Consecutive binding of IL-1α and IL-1β to ABT-981. To generate this representative sensogram, ABT-981 was immobilized on a Biacore sensor chip via goat anti-human IgG and 500 nM human IL-1β was injected followed by injection of 500 nM human IL-1α. No dissociation time of the IL-1β was allowed prior to the injection of the IL-1α. The sensogram indicates that human IL-1β binds to ABT-981, and IL-1α variable domains are still accessible for binding to IL-1α. The reciprocal of this study (binding of IL-1α followed by IL-1β) showed identical results (data not shown). The Y-axis of the plot is response units and the X-axis is time in seconds.

To determine if ABT-981 could bind IL-1α and IL-1β at the same time, a study was conducted using SPR (Biacore). Following the immobilization of ABT-981 on a sensor chip, the IL-1 antigens were injected one after another under saturating conditions without allowance of any dissociation phase. In one study, human IL-1α was injected over captured ABT-981 and immediately followed by injection of human IL-1β; no dissociation time was allowed before the human IL-1β injection. In another study, human IL-1β was injected over captured ABT-981 and immediately followed by injection of human IL-1α; no dissociation time was allowed before the human IL-1α injection. The shape of the sensograms and the binding responses were analyzed and the stoichiometry of the binding was calculated (**Fig. 3**). The stoichiometry indicated that between 1.5–2 molecules of both human IL-1α and IL-1β can bind to each ABT-981 DVD-Ig (**Table 4**). Overall, these data demonstrate that human IL-α binds to both ABT-981 binding domains even after both human IL-1β binding domains are saturated, and vice versa. These data imply that ABT-981 is physically capable of simultaneously binding 2 human IL-α and 2 human IL-1β molecules.

**Table 4.** Apparent stoichiometry for consecutive binding of IL‑1α and IL‑β to ABT-981

First injection	Stoichiometry	Second injection	Stoichiometry
Human IL-1α	1.8	Human IL-1β	1.6
Human IL-1β	1.8	Human IL-1α	1.7

### Specificity of ABT-981

To define the specificity of ABT-981, proteins with homology to human IL-1α and IL-1β were identified using NBCI Blast, and any proteins having 28% or greater homology to human IL-1α or IL-1β were selected and defined as “members of the IL-1 family." These proteins were IL-1ra/IL-F3, IL-18/IL-1F4, IL-36Ra/IL-1F5, IL‑36α/IL‑1F6, FIL1ζ /IL-1F7, IL-36β/IL-1F8, IL-36γ/IL-1F9, and IL-1F10. Recombinant versions of each of these 10 IL-1 family members were generated at AbbVie or purchased commercially, and used in an SPR binding study to determine if ABT-981 binds to IL-1 family members in addition to IL-1α and IL-1β. Multiple SPR experiments using 500 nM of each IL-1 family member were carried out to assess binding to immobilized ABT-981.

**Table 5** summarizes the results of the experiments evaluating ABT-981 binding to IL-1 family members. Positive binding events are represented as “+” and the absence of a binding response is represented as “−." Overall, the only IL-1 family members tested that bound to ABT-981 in the SPR format were IL-1α and IL-1β, indicating that ABT-981 is highly specific for its intended targets.

**Table 5.** Specificity of ABT-981 to human IL-1α and IL-1β

Ligand	Binding to ABT-981
FIL1ζ /IL-1F7	−
IL-36Ra/IL-1F5	−
IL-36β/IL-1F8	−
IL-36α/IL-1F6	−
IL-36γ/IL-1F9	−
IL-1Ra/IL-1F3	−
IL-1F10	−
IL-18/IL-F4	−
IL-1α	+
IL-1β	+

### In vivo pharmacologic activity of ABT-981

**Figure 4.**
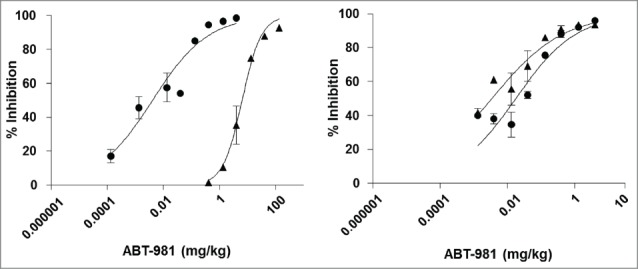
In vivo neutralization of human and cynomolgus IL‑1α (left panel) and IL‑1β (right panel) with ABT‑981. A pharmacodynamic mouse model was established to demonstrate in vivo activity of ABT-981. Upon injection with 30 ng of either human (•) or cynomolgus (5) IL‑1α and IL‑1β, mouse IL-6 was measured in serum. The loss of IL-6 in serum was correlated to the dose of ABT-981 given to each animal and a percent inhibition by ABT-981 was calculated.

The in vivo activity of ABT‑981 was evaluated by testing its ability to block exogenously administered recombinant human IL-1α- or IL-1β-induced IL-6 production in mice. A 30 ng dose of exogenously administered human IL-1α or IL-1β was selected based on the level of induction of IL-6 that could be accurately measured from female C57/BL6 mouse serum and that provided an adequate signal-to-background ratio to demonstrate neutralization by ABT-981 (data not shown). Female C57/BL6 mice were injected intraperitoneally (i.p.) with phosphate-buffered saline (PBS) or ABT-981 on day 0. On day 1, each mouse was injected with PBS or 30 ng of human or cynomolgus IL-1α or IL-1β. Two hours following injection of IL-1, levels of murine IL-6 in plasma were measured and data were plotted as percent inhibition (**Fig. 4**). These data show that following administration of ABT-981, levels of murine IL-6 generated in response to exogenously administered human and cynomolgus IL‑1α and IL-1β decreased in a dose-dependent manner, and the individual and average ED_50_ and ED_80_ values generated from 2 independent experiments are shown in **Table 6**. Overall, the dose of ABT-981 required to neutralize the effects of 30 ng of human or cynomolgus IL-1β was comparable within a 3-fold range (ED_80_ range from 0.094 to 0.28 mg/kg). In comparison, the dose of ABT‑981 required to neutralize the effects of 30 ng of cynomolgus IL-1α was over 170-fold greater compared to the dose required to neutralize the effects of 30 ng of human IL‑1α (ED_80_ of 18.2 mg/kg for cynomolgus IL-1 α compared to 0.104 mg/kg for human IL-1α), consistent with the lower in vitro affinity and potency of ABT-981 to cynomolgus IL-1α (**Table 3**). From this pharmacological study, complete neutralization of cynomolgus IL-1α would theoretically be achieved in vivo at doses >18 mg/kg, and complete neutralization of cynomolgus IL-1β at doses >0.09 mg/kg, enabling assessment of the mechanistic toxicity of ABT-981 in cynomolgus monkeys.

**Table 6.** ED_50_ and ED_80_ for ABT‑981 to neutralize human or cynomolgus IL‑1α or IL‑1β

	Human IL-1α		Cynomolgus IL-1α		Human IL-1β		Cynomolgus IL-1β	
	ED_50_ (mg/kg)	ED_80_ (mg/kg)	ED_50_ (mg/kg)	ED_80_ (mg/kg)	ED_50_ (mg/kg)	ED_80_ (mg/kg)	ED_50_ (mg/kg)	ED_80_ (mg/kg)
Expt #1	0.007	0.17	5.2	16.15	0.02	0.27	0.002	0.098
Expt #2	0.002	0.04	7.7	20.16	0.012	0.32	0.006	0.11
Average	0.005	0.104	6.5	18.2	0.016	0.28	0.004	0.094

### Single-dose pharmacokinetics of ABT-981 in mouse, rat and cynomolgus monkey

**Figure 5.**
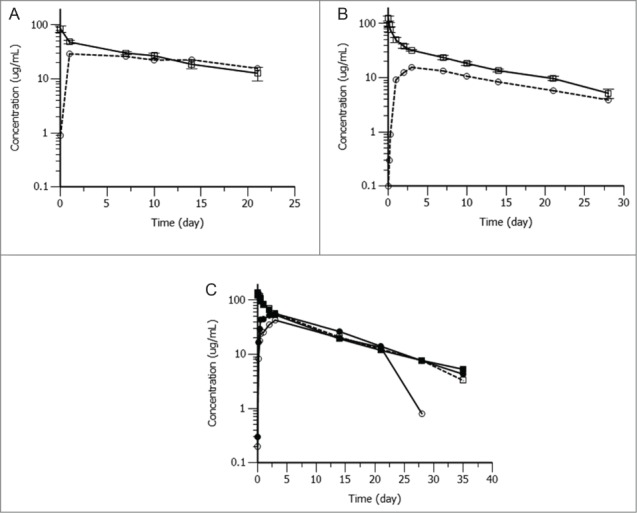
(**A**) Mean (±SD) serum concentrations of ABT-981 following a 5 mg/kg intravenous () or subcutaneous () dose in male BALB/c mice. (**B**) Mean (±SD) serum concentrations of ABT-981 following a 4 mg/kg intravenous (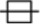) or subcutaneous (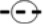) dose in male Sprague‑Dawley rats. (**C**) ABT-981 serum concentrations following a single 5 mg/kg intravenous (Monkey 1: 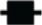 and Monkey 2 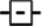) or subcutaneous (Monkey 3: 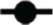 and Monkey 4: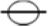) dose in female Cynomolgus monkeys (C).

The pharmacokinetic properties of ABT-981 were assessed following single IV or SC doses of 5 mg/kg in male BALB/c mice, 4 mg/kg in male Sprague-Dawley rats, and 5 mg/kg in female cynomolgus monkeys (**Table 7**). After IV dosing in BALB/c mice, ABT-981 exhibited low clearance (0.27 mL/h/kg), a small volume of distribution (95 mL/kg), and a long half-life of 10.5 d. After SC administration of ABT-981 in mice, the C_max_ was 29.2 μg/ml, the half-life was 20.3 d and bioavailability was ∼100% (**Fig. 5A**). In 2 of 6 animals in the IV dose group and in 5 of 6 mice receiving an SC dose, serum concentrations of ABT-981 declined rapidly after 7–10 days, probably due to the animals developing an anti-drug antibody (ADA) response (for individual animal data, see **Fig. S2**). The animals showing probable ADA responses were eliminated from the pharmacokinetic calculations.

**Table 7.** Summary of main pharmacokinetic parameters of ABT-981 following a single intravenous or subcutaneous dose in BALB/c mouse, Sprague‑Dawley rat and cynomolgus monkey

Intravenous Dose							
Species	Gender: n^§^	Dose (mg/kg)	T½° (day)	CL (mL/hr/kg)	V_z_ (mL/kg)	V_ss_ (mL/kg)	AUC_0-∞_ (mg·hr/mL)
Mouse	Male: n = 4[6]	5	10.5	0.27 (0.06)	99 (8)	95 (6)	19.4 (3.7)
Rat	Male: n = 5[5]	4	10.0	0.28 (0.03)	96 (6)	86 (4)	14.5 (1.3)
Monkey	Female: n = 2[2]	5	10.4	0.22, 0.22	74, 86	55, 66	22.8, 22.8
Subcutaneous Dose							
Species	Gender: n^§^	Dose (mg/kg)	T½ (day)	T_max_ (day)	C_max_ (μg/mL)	F (%)	AUC_0-∞_ (mg·hr/mL)
Mouse	Male: n = 1[6]	5	20.3	1.0	29.2	∼100	22.5
Rat	Male: n = 1[5]	4	12.0	3.0	16.0	52	7.5
Monkey	Female: n = 1[2]	5	8.0	3.0	57.0	95	21.6

° harmonic mean; mouse and rat IV data provided as Mean (SD).

^§^n = x[y] number of animals included in pharmacokinetic calculations [total number of animals dosed]. Animals with ADA responses were omitted from pharmacokinetics calculations.

Abbreviations: T_1/2_ – half-life; CL – clearance, V_z_ – volume of distribution at terminal phase; V_ss_ – volume of distribution at steady state; AUC – area under the curve from time 0 to infinity; T_max_ – time to reach C_max_; C_max_ – maximum serum concentration after administration; F – bioavailability.

After IV dosing in Sprague-Dawley rats, ABT-981 exhibited low clearance (0.28 mL/h/kg), small volume of distribution (86 mL/kg), with long half-life of 10.0 d (**Fig. 5B** and **Table 7**). Following SC administration in rat, a C_max_ of 16.0 μg/ml, with a half-life of 12.0 d and 52% bioavailability was observed (**Fig. 5B**). One of 6 rats in the IV dose group and 5 of 6 rats receiving a SC dose exhibited a drop in serum concentration after 10–14 d likely due to ADA, therefore these animals were eliminated from pharmacokinetic calculations.

After IV dosing in cynomolgus monkeys, ABT-981 exhibited low clearance (0.22 mL/h/kg), small volume of distribution (61 mL/kg) and long half-life of 10.4 d. After SC administration, the half-life of ABT-981 was 8.0 d and bioavailability was 95% (**Fig. 5C**). In the SC dose group, serum concentrations declined after Day 20 in 1 of the 2 monkeys, and this animal was eliminated from the pharmacokinetic calculations.

### Biophysical characterization and stability assessment of ABT-981

**Figure 6.**
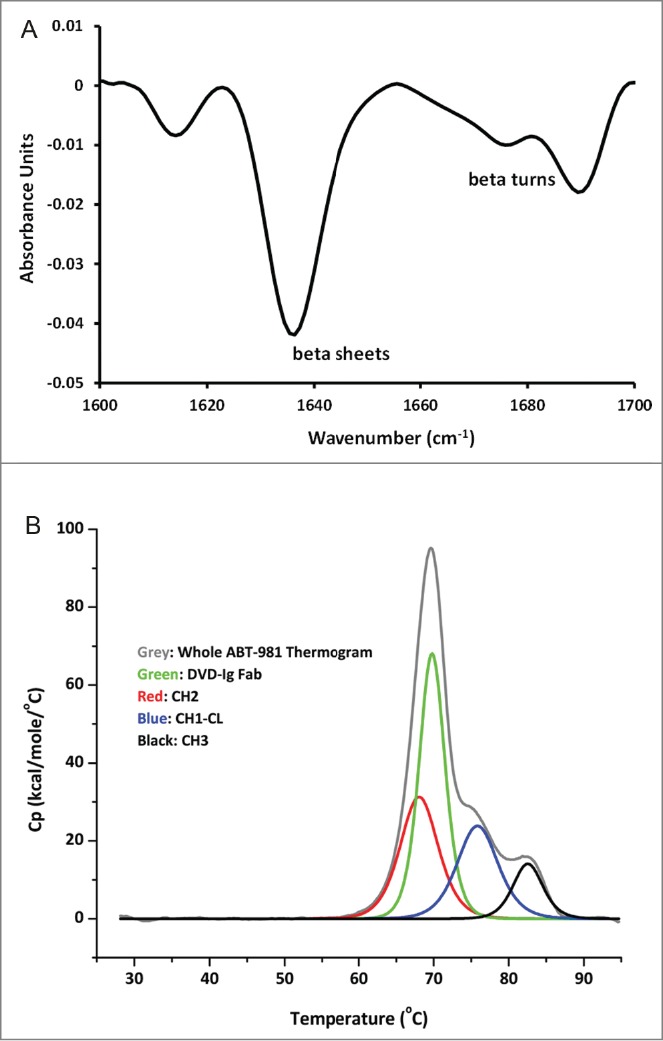
Biophysical characterization of ABT-981 in solution. (**A**) Fourier transform infrared spectroscopy chromatogram for secondary structure characterization indicating ABT-981 is comprised primarily of beta sheets supplemented by beta turns. (**B**) Differential scanning calorimetry thermogram following thermal stability analysis. The unfolding transitions for the individual domains of ABT-981 are assigned according to the legend.

To define the biophysical properties of ABT-981, the following analyses were completed: secondary structure, thermal stability, and short-term accelerated stability. Secondary structure determination by Fourier transform infrared spectroscopy of a 1 mg/mL ABT-981 sample produced the chromatogram shown in **Figure 6A**. A prominent peak comprising 57% of the total area was observed at ∼1638 cm^−1^ and ranged from 1627 to 1642 cm^−1^. This indicates that ABT-981 is composed primarily of beta-sheets supplemented by beta-turns.^46^ The lack of any appreciable signal at 1656 cm^−1^ indicates the absence of alpha helixes. The thermal stability of a 1 mg/mL ABT-981 sample was assessed using differential scanning calorimetry (DSC). The sample was heated at a 1°C/minute scan rate from 25°C to 95°C, and **Figure 6B** shows the thermogram following the analysis. The large peak at 69°C (in green) likely corresponds to the unfolding transition of the ABT-981 DVD-Ig Fab fragment consisting of the outer and inner variable domains. Overall, ABT-981 begins to unfold at ∼55°C, which is similar to the onset of unfolding temperature for IgG1 molecules. The transitions of the constant heavy chain 1 / constant light chain domain (C_H_1-C_L_) and the C_H_3 domain (in blue and in black, respectively) are also similar to those observed for IgG1 antibodies.^47^ The enthalpy of the C_H_2 transition (red) may be slightly greater than expected due to the uncertainty of the deconvolution of its corresponding peak caused by the overlap with the DVD-Ig Fab peak.

Short-term accelerated stability studies were carried out on 100 mg/ml solutions of ABT-981 to define the physical and chemical stability of the molecule. Samples of ABT-981 were stored at 5°C (storage condition) and 40°C (stress condition) and were analyzed at regular intervals by size exclusion chromatography (SEC) and weak cation exchange chromatography (IEC). After two months of storage at 5°C, ABT-981 showed no difference in the distribution of size species (97.6% monomer at time zero vs. 97.1% at 2 months). However, a slight increase in basic species was observed (from 16.4% to 21.4%) with a concomitant loss in the main species (73.8% to 68.0%), while the acidic species distribution remained similar (9.8% to 10.6%). After two months of storage at 40°C, ABT-981 displayed an apparent loss in monomer species (97.7% to 87.3%) with increases in both aggregates (1.1% to 5.9%) and fragments (1.2% to 6.7%).

Although characterization studies were completely enabled with ABT-981 expressed from HEK293 cells, planned clinical studies required ABT-981 to be stably expressed from mammalian host cells. Several stable cell lines were engineered to express ABT-981, and one was selected to generate material for clinical studies because it expressed greater than 1 g/L of ABT-981 in standard bioreactor conditions (data not shown). In addition, stable cell-line-generated ABT-981 could routinely be purified to > 98% monomer using a protein A purification process and clearly produced the expected 63 kDa heavy chain band and the 36 kDa light chain band following SDS-PAGE analysis (data not shown).

## Discussion

Here, we describe the generation and characterization of ABT-981, a human dual-variable-domain immunoglobulin (DVD-Ig) that is a potent and specific neutralizer of both human IL-1α and IL-1β. The format and composition of this molecule was inspired by a series of studies demonstrating the feasibility of human and murine DVD-Ig generation,^40,48^ the relevance of inhibition of both mouse IL-1α and IL-1β in reducing OA progression and pain,^41^ ^42^ and improvement attributes desired in a therapeutic entity based on OA clinical trial data from other IL-1 inhibitors.^37–39^ The human antibody variable domains necessary to construct a human DVD-Ig with drug-like properties were identified, and were synthesized and characterized to allow the generation of several anti-IL-1α and IL-1β pilot DVD-Igs. A single human mAb to IL-1α (X3) was identified and characterized, and a humanized mAb to IL-1β (SK48-E26) was generated, characterized, and affinity matured using yeast surface display technology. Both mAbs showed suitable cross-reactivity to cynomolgus monkey IL-1α and IL-1β, which is important to enable toxicological studies, but did not cross-react to either mouse or rat orthologs.

Following the identification and characterization of human variable domains, human DVD-Igs were assembled by fusing heavy and light chain variable domains from each mAb via human linker amino acid sequences derived from human immunoglobulin hinge region amino acids. In general, the fusion orientation of any 2 variable domains has to be experimentally derived to enable generation of a DVD-Ig with appropriate potency, affinity, and drug-like properties. The orientation of the “outer” and “inner” human heavy- or light-chain variable domains was explored in some molecules to define which architecture resulted in the desired affinity and potency. In the case of the IL-1α mAb X3 and the affinity-matured IL-1β mAb E26.13 or E26.35, the optimal orientation was determined to be an architecture wherein the IL-1β neutralizing domain was in the “outer” position and the IL-1α neutralizing domain was in the “inner” position, with both LL and SS linker sequences. In each case, these human DVD-Igs were expressed with an IgG1/k constant region containing IgG1 amino acid hinge region mutations to eliminate effector functions by altering interactions with Fcγ receptors. These mutations occur at positions (L234A, L235A) in an IgG1 molecule; ^49,50^ however, the corresponding hinge domain mutations for a DVD-Ig are located at positions (L364A, L365A). The DVD-Ig referred to as E26.13-SS-X3 had potency and affinity characteristics comparable to, and in many cases improved characteristics, compared to the mAbs from which it was engineered, indicating that the “outer” domain of the DVD-Ig architecture was ideal for the expression and function of the IL-1β neutralizing domain and the “inner” domain was ideal for the expression and function of the IL-1α neutralizing domain. The E26.13-SS-X3 DVD-Ig was re-named ABT-981.

Using SPR (Biacore) technology, we demonstrated that ABT-981 is capable of simultaneously binding 2 human IL-1α and 2 human IL-1β molecules. Analysis of data following an additional SPR binding study indicated that ABT-981 binds only to human IL-1 family members IL-1α and IL-1β, and not to other IL-1 family members, including IL‑18/IL-1F4, IL-1Ra/IL-1-F3, IL-36Ra/IL-1F5, IL-36α/IL-1F6, FIL1ζ /IL-1F7, IL‑36β/IL-1F8, IL-36γ/IL-1F9, and IL-1F10.

Because ABT-981 does not cross-react with rodent IL-1α- or IL-1β, established rodent models were not suitable for the characterization of the pharmacological properties of ABT-981 in vivo. As a result, in vivo studies were completed using exogenously-administered recombinant human IL-1α- or IL-1β-induced IL-6 production in mice. The study demonstrated that ABT-981 is stable in rodent serum following administration, and neutralizes human IL-1α and IL-1β in vivo as indicated by the decrease in mouse IL-6 production. Given the lower affinity and potency of the anti-IL-1α antibody variable domains (derived from mAb X3) to cynomolgus IL-1α, it was important to estimate doses of ABT-981 required to completely neutralize cynomolgus IL-1α to enable a toxicological evaluation of ABT-981. ABT‑981 inhibited IL-6 production resulting from exogenously-administered human and cynomolgus IL-1α and IL-1β in a dose-dependent manner. It is not well understood why the affinity and potency of ABT-981 is significantly less on cynomolgus IL-1α compared to human IL-1α, but one hypothesis is that there are residues in the cynomolgus ortholog that are unique from human within the epitope recognized by ABT-981 (and specifically, the X3 human autoantibody mAb that was used to generate the ABT-981 DVD-Ig).

Finally, the pharmacokinetic properties of ABT-981 were assessed following single IV or SC administration in BALB/c mice, Sprague-Dawley rats and cynomolgus monkeys. After IV dosing, ABT-981 exhibited low clearance, with low volumes of distribution, and long half-lives of ∼10 d in all 3 species. Following SC administration, bioavailability was variable from 52% in rat to > 95% in mouse and monkey. Serum elimination half-lives following the SC dose were long at 20.3 d in mice, 12 d in rat and 8.0 d in monkeys. Some animals exhibited reduced serum exposure 7–10 d after dosing, mostly in the subcutaneous dose groups. These animals were assumed to have developed an anti-drug antibody (ADA) response; however no specific evaluations were undertaken to measure ADA levels or to determine which epitopes on ABT-981 were associated with the probable response. The overall pharmacokinetic properties of ABT-981 in mouse, rat and monkey indicate that the human DVD-Ig architecture displays typical therapeutic antibody-like pharmacokinetic properties despite its size and complexity and is a bona fide new molecular entity suitable for clinical development.

The secondary structure of ABT-981 is primarily comprised of beta sheets with accompanying beta turns, and no alpha helices were observed. This finding is expected of an IgG1-based molecule and indicates that the DVD-Ig, despite being an artificially engineered IgG1 protein, still retains the proper secondary structure of its parental antibody components. The observation that the thermal stability of ABT-981 is comparable to an IgG1 molecule in solution is unexpected because its outer variable domain is not adjacent to the C_H_1-C_L_ region, a domain known to stabilize the V_H_-V_L_ in the immunoglobulin format.^51^ The intact beta sheet secondary structure and high thermal stability of ABT-981 is reflected in its stability during storage at 100 mg/ml, especially under stress conditions of 40°C, which show low levels of physical and chemical degradation.

Overall, the potency, affinity, specificity, stability, and pharmacokinetics demonstrated by ABT-981 make it a suitable therapeutic protein to manufacture using mammalian host cells. ABT-981 has recently been investigated in Phase 1 clinical studies, where it exhibited acceptable pharmacokinetic, tolerability, and safety profile in healthy volunteers and patients with OA of the knee.^52,53^ These studies support further investigation of ABT-981 in an OA population in Phase 2 trials, and possibly in other IL-1 mediated disease populations as well.^54^

## Materials and Methods

### Generation of affinity-matured humanized IL-1β antibodies

V_H_ and V_L_ amino acid sequences of a humanized SK48-E26 antibody specific for human IL-1β^44^ were used to construct libraries for affinity maturation by yeast surface display. Three scFv antibody libraries containing variant V_H_ or V_L_ sequences were generated in yeast at CDR residue positions that are mutated at higher frequencies in human antibodies. The limited mutagenesis was introduced by polymerase chain reaction using oligonucleotide primers that contained 70% parental nucleotide sequence and 10% each of the other 3 nucleotides at codon positions (Kabat numbering) indicated in each library. Library 1 contained variant V_H_ in the CDRH1 and CDRH2 at positions 31, 33, 50, 52a, 55, 56, 57, 58, 60 and 3 sequence toggles at position 23(A/S), 24(A/S), and 62(T/S) to allow sequence germ-lining during library selection. Library 2 contained variant V_H_ in the CDRH3 at positions 95, 96, 97, 98, 99, 100, 100a, 100b, 102, and framework sequence toggles at position 84(P/A), 88(G/A), 91(F/Y), and 108(P/L). Library 3 contained variant V_L_ in CDRL1 at positions 30, 31, 32; CDRL2 at positions 50, 53, 55, 56; CDRL3 at positions 92, 93, 94, 96, and 97. Each yeast library was created using transformation methods described previously.^55^

Library affinity selections (equilibrium, on- and off-rate) were performed in the presence of biotinylated IL-1β (AbbVie) using magnetic bead sorting and fluorescence activated cell sorting. At least 2 rounds of selection based on magnetic bead enrichment was performed, followed by at least 3 rounds of screening based on fluorescence activated cell sorting ^56^ to identify variants having improved binding compared to the parental SK48-E26 antibody. A proportion of the isolated clones were sequenced to obtain 89 unique V_H_ and 41 V_L_ variant sequences. These sequences provided guidance to generate 2 additional yeast libraries, library 4 containing variant CDR sequences in the 3 V_H_ CDRs, and library 5 containing variant sequences in all 6 V_H_ and V_L_ CDRs. Affinity selections of these scFv libraries were performed in the presence of biotinylated human and cynomolgus IL-1β (AbbVie) using more stringent affinity selection conditions, including reduced incubation times of the biotinylated IL-1β and the libraries, increased incubation temperature of the biotinylated IL-1β and the libraries, and decreased biotinylated IL-1β concentrations incubated with the libraries. Upon completion of library selections, 8 variant scFv sequences were selected and expressed as human IgG1/k proteins (E26.1, E26.2, E26.11, E26.12, E26.13, E26.13, E26.35, E26.37) for further characterization.

### Antibody or DVD-Ig binding measurement by ELISA

To quantitate the binding of mAbs or DVD-Igs to IL-1α or IL-1β, 96-well ELISA plates were incubated overnight at 4°C with anti-human Fc antibody (Jackson Immunoresearch, 109-516-008) diluted in Pierce Buffer Superblock (Thermo Scientific, 37515) at a final concentration of 2 μg/mL. Plates were washed 5 times in washing buffer (PBS containing 0.05% Tween 20), and blocked for 1 hour at 25°C with 200 μL per well with Pierce Buffer Superblock. Blocking buffer was removed, and mAbs or DVD-Igs were added to each well at a final concentration of 2 μg/mL following dilution of each in 1xPBST (PBS containing 10% Pierce Buffer Superblock and 0.5% Tween-20). 100 μL of each mAb or DVD-Ig was added to the wells, followed by a 1 hour incubation at 25°C. The plates were washed 5 times in 1xPBST. Following this, 100 μL biotinylated human IL-1α or IL-1β was added to each well, at concentrations ranging from 1000 ng/mL to 0.597 pg/mL (achieved by preparing 1:6 serial dilution titrations with 1xPBST) followed by a 1 hour incubation at 25°C. The wells were washed 5 times in 1xPBST, then 100 μL of peroxidase-labeled streptavidin (KPL, 71–00–38) was added to each well and incubated for 1 hour at 25°C. Following this, plates were washed 5 times in 1xPBST, and 100 μL of Ultra-TMB ELISA (Thermo Scientific, 34028) was added to each well. Following color development, the reaction was stopped with 1 N HCl and absorbance at 450 nM was measured using a Molecular Devices plate reader and Softmax data analysis to generate average EC_50_ concentrations of biotinylated human IL-1α or biotinylated human IL-1β.

### Antibody or DVD-Ig potency by MRC-5 cell bioassay

MRC-5 cells acquired from ATCC (CCL^-^171) were cultured in DMEM (Life Technologies, 11090–81) containing 10% fetal bovine serum (Thermo Scientific, SH30070.03) plus the following supplements (all from Life Technologies): 1% L-glutamine (25030–081), 1% sodium bicarbonate (25080–094), 1% sodium pyruvate(11360–070), 1% non‑essential amino acids (11140–050), 50 units/mL penicillin and 50 μg/mL streptomycin (15140–122).

Cells were grown at 37°C in a 5% CO_2_ incubator. Twenty-four hours prior to the bioassay, 100 μL of MRC-5 cells were plated at a concentration of 1 × 10^5^/mL in a 96-well assay plate. On the day of the assay, 50 μL of human or cynomolgus IL-1α or IL-1β (50 pg/mL final concentration) was added to each well of a separate 96-well preparation plate. Antibodies or DVD-Igs (50 μL) were added to each well (1 × 10^−7^ to 1 × 10^−15^ M final concentration range) of the preparation plate and incubated with IL-1α or IL-1β for 1 hour at 37°C, 5% CO_2_. The IL-1α or IL-1β + antibody complexes (100 μL) were then removed from the preparation plate and added to the MRC-5 cells, and plates were incubated for 16–20 hours at 37°C in a 5% CO_2_ incubator. Human IL-8 production in cell supernatants was measured using a human IL-8 ELISA kit (R&D Systems, D8000C). Neutralization potency was determined by calculating average percent inhibition relative to the IL-8 release generated following stimulation with IL-1β or IL-1α alone. IC_50_ values were calculated using GraphPad Prism software.

### Antibody or DVD-Ig affinity binding measurement by surface plasmon resonance

The binding affinity of human antibodies to human and cynomolgus IL-1α and IL-1β was determined by SPR-based measurements with a Biacore® 3000 instrument (Biacore® AB) using running buffer HBS-EP (10 mM HEPES [pH 7.4], 150 mM NaCl, 3 mM EDTA, and 0.005% surfactant P20) at 25°C. Approximately 5000 RU of goat anti-human IgG Fc polyclonal antibody (Thermo Scientific, PI-31163) diluted in 10 mM sodium acetate (pH 4.5) was directly immobilized across a CM5 research grade biosensor chip using a standard amine coupling kit according to manufacturer's instructions and procedures at 25 μg/mL. Unreacted moieties on the biosensor surface were blocked with ethanolamine, and modified carboxymethyl dextran surface in flowcell 2 and 4 was used as a reaction surface. The association and dissociation rate constants for all Igs, k_on_ (unit M−1s−1) and k_off_ (unit s−1) were determined under a continuous flow rate of 25 μL/min. Rate constants were derived by making kinetic binding measurements of immunoglobulins using IL-1 concentrations ranging from 10–200 nM. Unmodified carboxymethyl dextran without goat anti-mouse IgG in flow cell 1 and 3 was used as the reference surface. The equilibrium dissociation constant (unit M) of the reaction between the immunoglobulins and the target antigen was then calculated from the average kinetic rate constants by the following formula: K_D_ = k_off_/k_on_. A similar protocol was used to measure the binding affinity of human DVD-Igs to human and cynomolgus IL-1 proteins. For kinetic analysis, rate equations derived from the 1:1 Langmuir binding model were fitted simultaneously to association and dissociation phases of all 8 injections (using global fit analysis) with the use of Biaevaluation 4.0.1 software. To determine the specificity of ABT-981 to IL-1 family members, a similar protocol was used with 10 different human IL-1 family member proteins injected at a concentration of 500 nM and a flow rate of 25 μL/min for 6 minutes over captured ABT-981. Evaluation of binding of IL-1 family members to ABT-981 was completed in triplicate within the same experiment and on the same chip surface. Recombinant human proteins were provided by the following sources: IL-1α (AbbVie), IL-1β (AbbVie), IL-1ra/IL-1F3, (R&D Systems, 280-RA-050), IL-18/IL-1F4 (AbbVie), IL-36Ra/IL-1F5 (R&D Systems, 1275-IL-025), IL-36α/IL-1F6 (R&D Systems 1078-IL-025), FIL1ζ /IL-1F7 (R&D Systems, 1975-IL-025), IL-36β/IL-1F8 (R&D Systems, 1099-IL-025), IL-36γ/IL-1F9 (R&D Systems, 2320-IL-025), IL-1F10 (Origene, TP313987).

### Construction of IL-1α/β DVD-Ig DNA expression plasmids

DVD-Igs were engineered by overlapping PCR amplification of antibody V_H_ and V_L_ amino acid cDNA sequences fused in-frame with intervening linker DNA sequences.^48^ DVD-Igs specific for IL-1α or IL-1β were generated using antibody variable domains from the human IL-1α autoantibody X3 ^43^ and affinity-matured versions of the IL-1β humanized antibody SK48-E26.^44^ DVD-Igs generated differed in the location of each variable domain within the DVD-Ig (outer vs. inner) and incorporation of the LL light chain linker (TVAAPSVFIFPP) or the LL heavy chain linker (ASTKGPSVFPLAP) to fuse the 2 variable domains on each chain. The amplified PCR products encoding full-length DVD-Ig heavy and light chains were subcloned into the pHybE expression vector^57^ and the open reading frame regions were confirmed by sequencing. Variable domain cDNA sequences in the DVD-Ig heavy chain were expressed with a human IgG1 constant domain containing leucine-to-alanine amino acid substitutions in the lower hinge region residues at positions 364 and 365 (EU numbering system positions 234 and 235) and variable domain cDNA sequences in the DVD-Ig light chains were expressed with a human kappa constant domain.

### Expression of IL-1α/β mAbs and DVD-Igs

Heavy chain- and light chain-expressing plasmids were expanded in *E. coli* and purified using Qiagen Hispeed Maxi Prep kits (Qiagen). HEK 293–6E cells were grown in FreeStyle293 + 25 μg/mL G418 + 0.1% Fluronic F-68 + 0.01 M HEPES media (Life Technologies) in sterile Erlenmeyer shake flasks with vent caps. The night before transfection, cells were seeded at 0.5–0.7 × 10^6^ cells/mL in a 500 mL flask containing 210 mL media, and cultures were maintained at 37°C, 5% CO_2_ in a humidified shaking incubator at ∼130 rpm for 16 hours. For each mAb or DVD-Ig, the heavy chain-expressing plasmid (40 μg) was mixed with the light chain-expressing plasmid (60 μg) in 10 mL transfection media (FreeStyle293 + 0.01 M HEPES) containing 500 μg linear 25 kDa PEI (Polysciences, 25439–2). The PEI+DNA mixture was allowed to incubate at room temperature for 15 minutes prior to addition to the cells. The PEI+DNA mixture was added to the cell flask with vigorous swirling, then cells were allowed to incubate at 37°C in a 5% CO_2_ humidified shaking incubator at 130 rpm for 24 hours. After 24 hours, 10% tryptone N1 media (tryptone w/v (TekniScience, 19553) dissolved into FreeStyle293 media) was added to the cell flask to a final v/v concentration of 0.5% to increase mAb or DVD-Ig expression levels. After tryptone N1 media addition, culture flasks were incubated at 37°C, 5% CO_2_ in a humidified shaking incubator at 130 rpm for 5–6 d.

### Purification of IL-1α/β mAbs or DVD-Igs

Cell supernatants containing mAbs or DVD-Ig were harvested and filtered through a 0.2 μM polyethersulfone filter. DVD-Igs were purified using protein A sepharose (PAS) affinity chromatography according to the manufacturer's instructions. Briefly, PAS (GE Healthcare Life Sciences, 17–5280–01) columns were equilibrated with protein A IgG binding buffer (Thermo Fisher Scientific, PI-21001), and cell supernatants loaded onto columns. Columns were washed with protein A IgG binding buffer and DVD-Igs were eluted off the affinity column in fractions by the addition of IgG elution buffer (Thermo Fisher Scientific, PI-21004). Fractions were neutralized with an alkaline buffer and fractions containing the most DVD-Ig (estimated by OD_280_) were pooled and subjected to dialysis into buffer pH 6.0. The final concentration of each purified DVD-Ig was estimated by OD_280_, and each was additionally characterized by SEC to ensure DVD-Igs were greater than 90% monomeric, and by mass spectrometry to confirm the expected molecular masses of the each DVD-Ig.

### ABT-981 consecutive antigen binding study by surface plasmon resonance

Goat anti-human IgG Fc was covalently linked to the carboxy methyl dextran matrix on the CM5 biosensor chip (Biacore AB, BR-1005–30) via free amine groups using an amine coupling kit and the immobilization wizard option in the Biacore T200 instrument's controlling software. Specifically, carboxyl groups of the dextran matrix on the chip were activated with 100 mM NHS and 400 mM EDC. Goat anti-human IgG (Pierce Biotechnology, PA1–85606) was diluted in 10 mM sodium acetate, pH 4.5 to a concentration of 25 μg/mL and was injected across the activated surface. Once the level of binding response reached the desired value, unreacted groups were deactivated by injection of 1 M ethanolamine. Approximately 10000 RU of goat anti‑human IgG Fc antibodies were immobilized on the chip surface. A modified CM surface coated with goat anti-human Fc antibody in Flowcell 1 was used as a reference surface. ABT-981 was diluted in HBS-EP (10 mM HEPES [pH 7.4], 150 mM NaCl, 3 mM EDTA, and 0.005% surfactant P20) (Biacore® AB, BR100188) to a concentration of 0.5 μg/mL and was injected over the goat anti-human IgG Fc surface on flow cell 3 at a flow rate of 5 μL/min or 10ul/min for 2 minutes to achieve a capture level of ∼241‑707 RU. First, human IL-1α was injected over captured ABT-981 (75 μL at a concentration of 500 nM) immediately followed by injection of human IL-1β (75 μL at a concentration of 500 nM), and both injections were performed at a flow rate of 30 μL/min. Human IL-1α was not allowed to dissociate before the human IL-1β injection in this study. Similarly, the design of the experiment was reversed by first injecting human IL-1β followed by human IL-1α to determine if the order of cytokine addition had any effect on these binding data. The stoichiometry of the binding was calculated according to the formula shown where RU_Ag_ represents apparent antigen binding response; RU_DVD_ represents DVD-Ig capture level (both in RU): Apparent Binding Stoichiomentry= (RU_Ag_ x MW_DVD_)/(RU_DVD_ × MW_Ag_).

### Assessment of in vivo neutralization activity of ABT-981

Female C57/BL6 mice aged 6–8 weeks old, and weighing ∼ 20 g (Jackson Laboratory) were housed 5 per cage. All animals were maintained at constant temperature and humidity under a 12 hour light/dark cycle and fed with normal rodent chow (Lab Diet 5010 PharmaServ) and water ad libitum. AbbVie is accredited by the Association for Assessment and Accreditation of Laboratory Animal Care International, and all procedures were approved by the AbbVie Institutional Animal Care and Use Committee prior to study initiation. For human IL-β and cynomolgus IL-β, groups of 5 mice each received i.p. injections on day 0 with 200 μL PBS or ABT-981 formulated in PBS to achieve the following dose groups in mg/kg: 4, 1.33, 0.4, 0.133, 0.04, 0.0133, 0.004, 0.0013. On day 1 (18 hours after injection of PBS or ABT-981), each mouse received injections of 100 μL PBS or 30 ng of human IL-β or cynomolgus IL-β formulated in 100 μL PBS. For human IL-α, groups of 5 mice each received i.p. injections on day 0 with 200 μL PBS or ABT-981 formulated in PBS to achieve the following dose groups in mg/kg: 4, 1.33, 0.4, 0.133, 0.04, 0.0133, 0.0013, 0.0001. On day 1 (18 hours after injection of PBS or ABT-981), each mouse received an injection of 100 μL PBS or 30 ng of human IL-α formulated in 100 μL PBS. For cynomolgus IL-α, groups of 5 mice each received an i.p. injection on day 0 with 200 μL PBS or ABT-981 formulated in PBS to achieve the following dose groups in mg/kg: 133, 40, 13.3, 4, 1.33, 0.4, 0.133, 0.0133. On day 1 (18 hours after injection of PBS or ABT-981), each mouse received an injection of 100 μL PBS or 30 ng of cynomolgus IL-α formulated in 100 μL PBS. For all groups, 2 hours following injection of human or cynomolgus IL-α, or human or cynomolgus IL-β, blood was collected from all animals and plasma was isolated to evaluate the levels of IL-6 using chemiluminecense (MSD, K151AKA-5). Levels of IL-6 measured in the presence of varying doses of ABT-981 were divided by levels of IL-6 measured following injection of PBS control and multiplied by 100 to generate percent inhibition values.

### Biophysical characterization and stability assessment

For secondary structure determination, thermal stability analysis, and short-term accelerated stability studies, samples of ABT-981 were prepared by dialysis into the appropriate formulation. ABT-981 solutions were filled into Slide-A-Lyzer dialysis cartridges (Thermo Scientific, 87729) and dialyzed into 2 L of the corresponding formulation buffer for 18–24 hours at room temperature with continuous stirring. The samples were retrieved from the cartridges and briefly passed through a 0.45 μm PVDF filter to remove any precipitation or particles. Protein concentrations were determined by UV absorbance measurements at 280 nm with a Cary 50 Bio UV-Visible spectrometer (Varian). When necessary, sample concentrations were adjusted with centrifuge spin filters (EMD Millipore, UFC903008) or by dilution to obtain the desired concentration.

Secondary structure was determined with a Tensor 27 Fourier transform infrared spectrometer equipped with a BIO-ATR II cell accessory and liquid nitrogen cooled mercury, cadmium, and telluride detector (Bruker). Blank formulation buffers and ABT-981 solutions were scanned along the spectral region of 900 to 4000 cm^−1^ and the signal from the blank formulation was subtracted from that of ABT-981. The second derivative of the resulting Fourier-transformed signal was obtained and the absorbance peaks from 1600 to 1700 cm^−1^ were examined based on published literature.^46^ Differential scanning calorimetry (DSC) samples were analyzed with a VP-Capillary DSC unit equipped with an autosampler (Microcal / Malvern). Thermograms of ABT-981 samples were obtained by injecting protein into the sample cell while blank buffer was placed into the reference cell. Solutions were heated at a 1°C/minute scan rate from 25°C to 95°C, using a fitting period of 16 seconds, a pre-scan wait time of 10 minutes, and measurements performed in none-feedback mode. Blank formulation thermograms were similarly obtained by injecting the solution into both the sample cell and the reference cell. Blank thermograms were then subtracted from that of the ABT-981 sample. The resulting thermogram was manipulated to obtain a flat baseline and subsequently fitted to a non-2 state model to obtain the unfolding transitions of the individual domains of ABT-981.

For short-term accelerated stability studies, ABT-981 at 100 mg/ml in a histidine, sucrose, polysorbate 80 formulation was sterile filtered and aliquoted into sterile 0.6 ml cyrovials (Corning, 30488) and tightly capped. Samples were stored at 5°C (storage condition) and 40°C (stress condition) and were subsequently analyzed at regular intervals by SEC and weak cation exchange chromatography (IEC). SEC was carried out on a TSKgel G3000SWXL column (5 μm, 7.8 mm ID × 30 cm length, TOSOH) connected to an Agilent HPLC unit equipped with a UV detector. Chromatograms were obtained at 214 nm with a flow rate of 0.25 mL/min and a mobile phase of 100 mM sodium phosphate + 200 mM sodium sulfate pH 6.8. IEC was carried out on a Pro-Pac WCX-10 analytical cation exchange column (Thermo Fisher Scientific, SP6703) connected to a similar HPLC unit.

### Single dose pharmacokinetic study of ABT-981 in mouse, rat, and cynomolgus monkey

ABT-981 was administered to male BALB/c mice, male Sprague-Dawley rats and female cynomolgus monkeys by slow IV bolus dose or SC injection at a 4 mg/kg (rats) or 5 mg/kg (mice and monkeys). Blood samples were collected from each animal over a period of 21 through 35 d post-dose, and incubated at room temperature for 2 hours to allow isolation of serum. All serum samples were stored at −80°C until analysis.

### Quantitation of ABT-981 in serum samples

Serum samples from mouse, rat, and monkey were analyzed using a chemiluminescence assay employing a biotinylated human IL-1β (AbbVie) for capture and sulfo-tag labeled goat anti-human antibody (MSD, R32AJ-1) for detection. The samples were diluted in assay buffer and the assay was carried out at a 1% final serum concentration according to the manufacturer's instructions. Briefly, the diluted serum samples were incubated with biotinylated human IL-1β (prepared in PBS) and sulfo-tag labeled goat anti-human mAb and were added to MSD streptavidin standard plates (Meso Scale Discovery, R32AD-5). After incubation for 1 hour with shaking (600 rpm) at 24°C, the plates were imaged using the MSD Sector Imager.

ABT-981 concentrations were determined with the help of a standard curve, and the lower limits of quantitation (LLOQ) were 0.1, 0.025 and 0.075 μg/mL for the mouse, rat and monkey studies, respectively. Standard curve fitting and data evaluation was performed using IDBS XLfit4 software with a 4-parameter logistic fit. Pharmacokinetic parameters for each animal were calculated using WinNonlin software Version 5.0.1 (Pharsight Corporation) by non-compartmental analysis using linear trapezoidal fit (NCA Models # 201 and 200 for IV and SC dosing, respectively).
