# Supplementary material for: Generation and characterization of ABT-981, a dual variable domain immunoglobulin (DVD-IgTM) molecule that specifically and potently neutralizes both IL-1α and IL-1β
Source: MAbs. 2015 Mar 12;7(3):605–19. doi: 10.1080/19420862.2015.1026501 (PMC4622731; doi:10.1080/19420862.2015.1026501)
Supplement: Supplemental_Material.zip [file kmab-07-03-1026501-s001.zip › Supplemental_Figure_Captions.docx]

**Supplementary Figure 1.** Alignment of affinity-matured SK48-E26 variant sequences. A. Sequence alignment of heavy chain variable domains (V_H_) from six SK48-E26 variants. Amino acids are numbered from 1 on the top line and from 61 on the bottom line (numbered according to the Kabat system). CDRs are underlined (CDR H1, H2, and H3) and residues modified by affinity maturation selections are shaded. B. Sequence alignment of light chain variable domains (V_L_) from two SK48-E26 variants. Amino acids are numbered from 1 on the top line and from 61 on the bottom line (numbered according to the Kabat system). CDRs are underlined (CDR L1, L2, and L3) and residues modified by affinity maturation selections are shaded. Each affinity-matured SK48-E26 variant antibody was generated by expression of the corresponding affinity-matured (V_H_) and the wildtype SK48-E26 (V_L_) or the indicated affinity-matured (V_L_).

**Supplementary Figure 2.** A. Pharmacokinetic profile of ABT-981 in 6 BALB/c mice following a 5 mg/kg IV administration. Symbols: Mouse 1 (
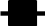
 ), Mouse 2 (
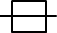
 ), Mouse 3 (
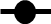
 ), Mouse 4 (
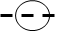
 ), Mouse 5 (
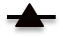
), Mouse 6 (
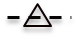
). Two animals exhibited reduced serum exposure approximately 15 days after dosing, likely due to ADA response.

B. Pharmacokinetic profile of ABT-981 in 6 BALB/c mice following a 5 mg/kg SC administration. Symbols: Mouse 1 (
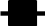
 ), Mouse 2 (
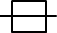
 ), Mouse 3 (
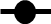
 ), Mouse 4 (
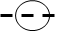
 ), Mouse 5 (
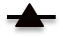
), Mouse 6 (
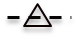
) Five animals exhibited reduced serum exposure approximately 7-10 days after dosing, likely due to ADA response.

C. Pharmacokinetic profile of ABT-981 in 5 SD rats following a 4 mg/kg IV administration. Symbols: Rat 1 (
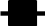
 ), Rat 2 (
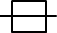
 ), Rat 3 (
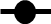
 ), Rat 4 (
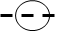
 ), Rat 5 (
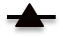
). D. Pharmacokinetic profile of ABT-981 in 5 SD rats following a 4 mg/kg SC administration. Symbols: Rat 6 (
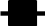
 ), Rat 7 (
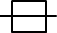
 ), Rat 8 (
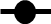
 ), Rat 9 (
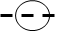
 ), Rat 10 (
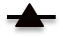
). Four animals exhibited reduced serum exposure approximately 10-14 days after dosing, likely due to ADA response.
