# Supplementary figures and images for: Generation and characterization of ABT-981, a dual variable domain immunoglobulin (DVD-IgTM) molecule that specifically and potently neutralizes both IL-1α and IL-1β
Source: MAbs. 2015 Mar 12;7(3):605–19. doi: 10.1080/19420862.2015.1026501 (PMC4622731; doi:10.1080/19420862.2015.1026501)

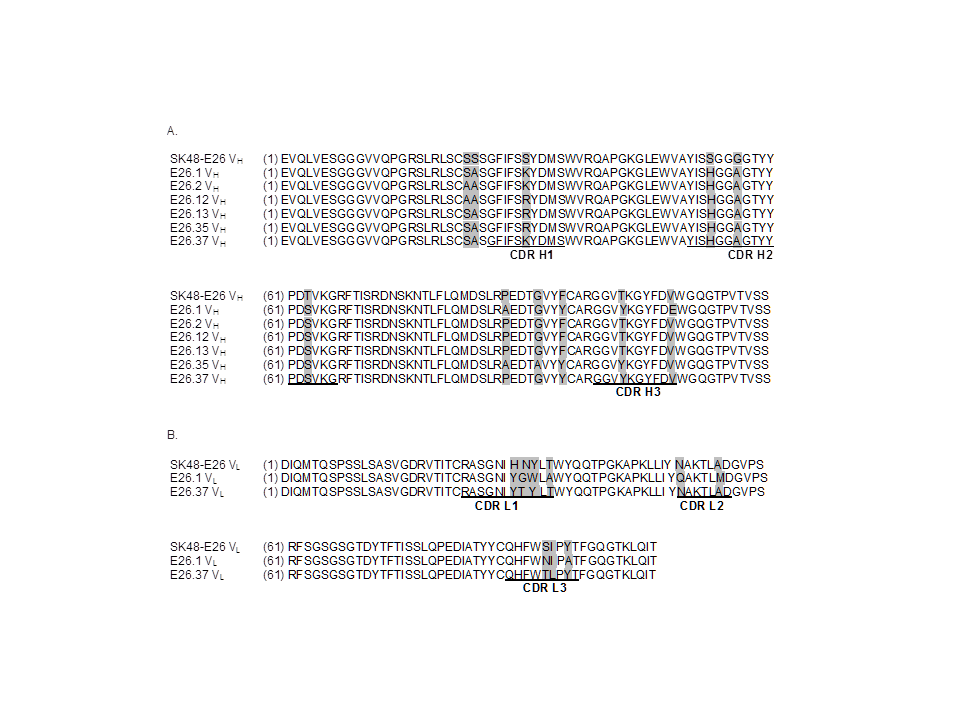

Supplement: Supplemental_Material.zip [file kmab-07-03-1026501-s001.zip › Supp Figure 1 ABT-981.tif]

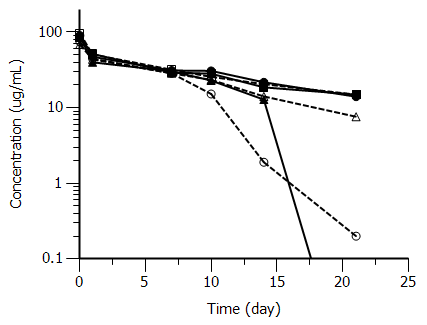

Supplement: Supplemental_Material.zip [file kmab-07-03-1026501-s001.zip › Supp Figure 2A ABT-981.tif]

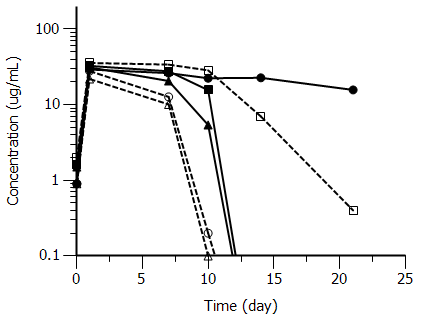

Supplement: Supplemental_Material.zip [file kmab-07-03-1026501-s001.zip › Supp Figure 2B ABT-981.tif]

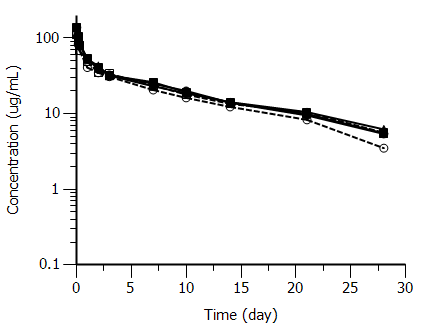

Supplement: Supplemental_Material.zip [file kmab-07-03-1026501-s001.zip › Supp Figure 2C ABT-981.tif]

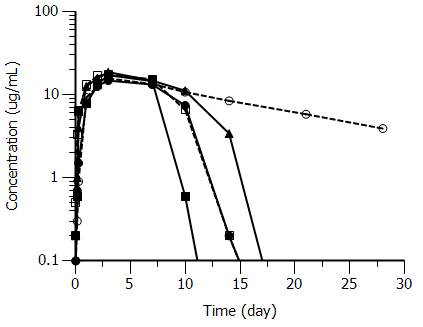

Supplement: Supplemental_Material.zip [file kmab-07-03-1026501-s001.zip › Supp Figure 2D ABT-981.tif]
